# A steroid receptor coactivator small molecule “stimulator” attenuates post-stroke ischemic brain injury

**DOI:** 10.3389/fnmol.2022.1055295

**Published:** 2022-12-01

**Authors:** Lisa K. McClendon, Roberto L. Garcia, Tyler Lazaro, Ariadna Robledo, Viren Vasandani, Zean Aaron Evan Luna, Abhijit S. Rao, Aditya Srivatsan, David M. Lonard, Clifford C. Dacso, Peter Kan, Bert W. O’Malley

**Affiliations:** ^1^Department of Molecular and Cellular Biology, Baylor College of Medicine, Houston, TX, United States; ^2^CoRegen, Inc., Baylor College of Medicine, Houston, TX, United States; ^3^Department of Neurosurgery, University of Texas Medical Branch, Galveston, TX, United States; ^4^Department of Neurosurgery, Baylor College of Medicine, Houston, TX, United States

**Keywords:** steroid receptor coactivator stimulation, transcriptional regulation, astrocytes, neuroprotection, cerebral ischemia, inflammation, oxidative stress

## Abstract

**Introduction:** Pathologic remodeling of the brain following ischemic stroke results in neuronal loss, increased inflammation, oxidative stress, astrogliosis, and a progressive decrease in brain function. We recently demonstrated that stimulation of steroid receptor coactivator 3 with the small-molecule stimulator MCB-613 improves cardiac function in a mouse model of myocardial ischemia. Since steroid receptor coactivators are ubiquitously expressed in the brain, we reasoned that an MCB-613 derivative (MCB-10-1), could protect the brain following ischemic injury. To test this, we administered MCB-10-1 to rats following middle cerebral artery occlusion and reperfusion.

**Methods:** Neurologic impairment and tissue damage responses were evaluated on day 1 and day 4 following injury in rats treated with control or 10-1.

**Results:** We show that 10-1 attenuates injury post-stroke. 10-1 decreases infarct size and mitigates neurologic impairment. When given within 30 min post middle cerebral artery occlusion and reperfusion, 10-1 induces lasting protection from tissue damage in the ischemic penumbra concomitant with: (1) promotion of reparative microglia; (2) an increase in astrocyte NRF2 and GLT-1 expression; (3) early microglia activation; and (4) attenuation of astrogliosis.

**Discussion:** Steroid receptor coactivator stimulation with MCB-10-1 is a potential therapeutic strategy for reducing inflammation and oxidative damage that cause neurologic impairment following an acute ischemic stroke.

## Introduction

Stroke is a pervasive disease worldwide, and a leading cause of morbidity and the fifth leading cause of death in the United States, with acute ischemic stroke (AIS) being the most common etiology (Goyal et al., 2016). Currently, the only FDA-approved medication for AIS is tissue plasminogen activator (tPA), which promotes clot degradation and reperfusion. In select patients with a large vessel occlusion, the addition of mechanical thrombectomy (MT) with thrombolysis with tPA has become the standard of care for revascularization (Albers et al., 2018; Nogueira et al., 2018). Nonetheless, AIS patients are still at an unacceptably high risk of death and disability (Albers et al., 2018; Nogueira et al., 2018) thus other therapies are sorely needed. While reperfusion focuses on restoring blood flow after AIS, neuroprotection refers to strategies that can reduce cerebral injury secondary to ischemia, but this currently remains an unmet clinical need.

While many other neuroprotective candidates have been explored in animal experiments and some in human trials (Saver et al., 2015; Hill et al., 2020), none have successfully improved AIS outcomes. Although they have largely been negative trials, these studies have provided valuable insights into how future trials might be designed to achieve better responses—specifically, therapies that appreciate the complexities of the ischemic cascade by affecting multiple injury response pathways. We recently discovered a small molecule stimulator of steroid receptor coactivators (SRC), called MCB-613 (Wang et al., 2015), that reduces ischemic injury after myocardial infarction by direct cardiomyocyte protection, mitigation of immune cell infiltration, and attenuation of pathologic fibroblast remodeling (Mullany et al., 2020). While the results of the study are very promising for cardioprotection, we considered this to also be a prime candidate for neuroprotection after AIS, as the heart and brain share many of the primary drivers of tissue damage after acute ischemic injury, including oxidative stress and inflammation.

SRCs are a family of nuclear proteins (SRC-1, -2, and -3) that are ubiquitously expressed and required for the transcription of ~80% of all genes (Lanz et al., 2010). As a result, SRC activation has been implicated in a broad array of cellular functions, including cell proliferation, regeneration, immune modulation, antioxidant defense, and angiogenesis (Lonard and O’Malley, 2007; Lanz et al., 2010). Our team has demonstrated that SRCs are extensive organizers of growth and repair since their discovery 27 years ago (Onate et al., 1995). For optimal tissue healing after injury, injury responses necessitate a strong transcriptome response coupled with cellular reprogramming including coordinating gene expression programs. After tissue injury, SRCs work to maintain cellular homeostasis by coordinating various gene expression programs including antioxidant-defense, cell survival, and angiogenesis (Lonard and O’Malley, 2007; Chen X. et al., 2010; Lanz et al., 2010; Chen et al., 2015). Thus, unlike single target therapies, the ability of SRCs to serve as coordinators of many wound healing gene expression programs predisposes SRCs to be ideal therapeutic targets for repair after ischemic injury. Moreover, the SRCs are expressed in all areas of the brain (Sun and Xu, 2020).

Considering SRC activation has been shown to beneficially regulate the drivers of cardiac ischemic injury in our previous experiments (Mullany et al., 2020), we tested the application of a more metabolically stable and potent MCB-613 derivative, 10-1, in a rat model for middle cerebral artery occlusion followed by reperfusion (MCAO R). We assessed the neurologic impairment and infarct size, and neuronal injury. We also assessed expression of key inflammatory, neuronal excitotoxicity, and oxidative injury markers.

## Materials and Methods

Animal procedures were approved under our Institutional Animal Care and Use Committee and conducted under National Institutes of Health guidelines for the Care and Use of Laboratory Animals. All experimental methods were standardized and performed by specified investigators to minimize potential confounders. All investigators were blinded throughout the experiment. Using computer-based randomization, rodents were assigned to the treatment groups prior to the initial procedure. Forty male Sprague Dawley rats (Charles River Laboratories), aged 7–9 weeks and weighing 225–250 grams, were divided into two experiments: 24-h (*n* = 20) and 4-day survival (*n* = 20). Due to their bigger size, only male mice were employed in this study in order to rule out the influence of gender and treatment response on stroke recovery. The subjects received equal volume intraperitoneal (IP) injections of either 10-1 (20 mg/kg) or saline control 30 min after reperfusion or sham surgery then injected daily if they survived for more than 24 h.

For the 24-h survival experiment, 10 rodents were randomly assigned to 10-1 and 10 rats were assigned to the control groups. In the 4-day experiment, 10 rodents were randomly assigned to the 10-1 and 10 to the control groups.

### Surgical preparation

Atropine sulfate (0.5 mg/kg IP), buprenorphine SR (1 mg/kg SQ), and meloxicam SR (2 mg/kg SQ) were injected 1 h before anesthesia. General anesthesia was induced using 5% isoflurane in 100% oxygen, by placing the rats in an induction chamber for approximately 3–5 min. The animals were then intubated with a 16-gauge angiocatheter and mechanically ventilated using a Harvard Apparatus VentElite ventilator. A surgical plane of anesthesia was maintained throughout the procedure with 2% isoflurane at 100% medical air. Rectal temperature, respirations, and pulse oximetry were monitored *via* an automated monitoring system (HPMS Model# 75-1501, Harvard Apparatus, Holliston, MA, USA). Body temperature was continuously monitored and maintained between 36.5 and 37°C throughout the procedure.

The scalp was shaved and cleaned using an iodine-based solution. The surgical field was draped with sterile linens. With the animal placed in a prone position, a 2–2.5 cm midline sagittal incision was performed and the scalp with periosteum was reflected to expose the left parietal bone. A laser doppler flowmetry (LDF) probe and holder were affixed to the left parietal bone using cyanoacrylate adhesive. The animal was then placed in a supine position for the remainder of the procedure.

### Middle cerebral artery occlusion

Following the methodology described in Longa et al. (1989), under microscopy, blunt dissection was performed through the carotid triangle to expose the left common carotid artery (CCA) and bifurcation of the external carotid artery (ECA) and internal carotid artery (ICA). The superior thyroid artery (STA) of the ECA was then isolated and coagulated, followed by the occipital artery. The ICA was carefully isolated from the adjacent vagus nerve. Next, two 3–0 silk sutures were placed around the ECA with one ligated distal to the STA. Cerebral blood flow (CBF) was monitored and recorded for baseline readings with the LDF system (moorVMS-LDF2, Moor Instruments) for 5 min. Two micro-vascular clips were placed on the CCA and ICA to prevent any bleeding, and the ECA was partially severed with microscissors. A 4–0 monofilament nylon suture with an occlusive diameter of 0.41 mm was inserted through the proximal ECA into the ICA, and the remaining ECA was severed. The micro-vascular clip on the ICA was removed and the filament was advanced until the LDF showed a 70% decrease from baseline CBF. The silk suture around the ECA stump was tightened around the intraluminal nylon suture to prevent bleeding. Occlusion time was set for 90 min then the filament was removed to allow for reperfusion.

### Exclusion criteria and stroke-related deaths

Criteria for exclusion consisted of excessive bleeding occurring during surgery, operation time exceeded 120 min, anesthesia recovery time exceeded 30 min, animals died prior to the scheduled euthanasia date or if subarachnoid hemorrhage was found during postmortem examination. Stroke-related deaths were defined as post-operative deaths due to neurologic devastation not associated with the above exclusion criteria. One animal was excluded in the 24 h cohort and six animals (four 10-1 and two control) were excluded in the 4 day cohort due to stroke-related death. No data were collected from these animals and there was no statistical difference due to stroke-related death.

### Neurologic assessment

A modified Bederson test was used to assess for neurologic impairments beginning on post-operative day 1. The scoring was as follows: (1) Forearm Flexion was graded 0–1; (2) resistance to Lateral Push was graded 0–2; and (3) circling Behavior was graded 0–3. The sum of these assessments determined the final score of 0 (normal) to 6 (severe) each day (17).

### Infarct volume measurements

Brains were sliced coronally into 2 mm slices and incubated at 37°C for 20 min in 2,3,5-triphenyltetrazolium chloride (TTC; Liu et al., 2009). Brains were then fixed in formalin and then paraffin was embedded. Brain slices were photographed and infarct volume was measured using Image J (NIH.gov). Infarct area was calculated by tracing unstained regions in each section (both sides) and multiplied by slice thickness to calculate infarct volume. For measurements of cortical and subcortical infarction sizes, hematoxylin and eosin (H&E) slides were made from paraffin embedded brain sections. Cortical and subcortical structures were consistently identified using the Rat Brain Atlas as a reference (Swanson, 2018).

### Immunofluorescence staining

Five micron sections were cut and placed onto slides. Immunofluorescence was performed by first removing the paraffin and then rehydrating the sections. After that, antigen retrieval was performed (Antigen unmasking solution, Tris-based, Vector Labs cat#H-3301). Sections were permeablized with 0.4% Tritonx-100-PBS, blocked with 10% normal goat serum (NGS) in PBS with 0.4% Triton X-100, and then incubated with primary antibody in blocking solution NeuN (1:200, Thermo Fisher, 26975-1-AP) ARG1 (1:200, Santa Cruz, SC-18354), GFAP (1:200, Invitrogen, MA5-12023), IBA1 (1:200, Invitrogen, MA5-41621) GLT-1 (1:200, AB1783, Sigma-Aldrich, NRF2 (1:200, Invitrogen, PA5-27882) followed by secondary (1:400 sary), and then DAPI (1:400 Thermo Fisher Scientific Cat#62248). Fluorescence was counted from three images per high-powered field (HPF) at 20× and quantified using ImageJ software. TUNEL stain was performed according to the manufacturer’s instructions (Sigma, ApopTag Fluoresce in *in situ* Apoptosis Detection Kit, S7100) and was detected with Cleaved Caspase-3 (Asp175) Antibody (Alexa Fluor^®^ 488 Conjugate, Cell Signaling cat #9669). *n* = 10 control and *n* = 10 drug treated brains at each time point.

### Quantification of microglial activation by cell shape

Microglia were visualized by immunohistochemical staining for Iba-1. ImageJ software analysis of three images of the penumbra region from three control and three drug-treated rats was used to determine cell body size.

### Statistics

Results are reported as the mean ± SEM. The statistical significance of the difference between means was assessed using IBM SPSS Software using the unpaired 2-tailed Student’s *t-*test (for the comparison of two groups). Pless than 0.05 was considered significant. With 10 animals per group, we had 80% power to detect a 1.3 standard deviation difference between the two groups.

## Results

### 10-1 reduces infarct size and neurologic impairment

**Figure 1:**
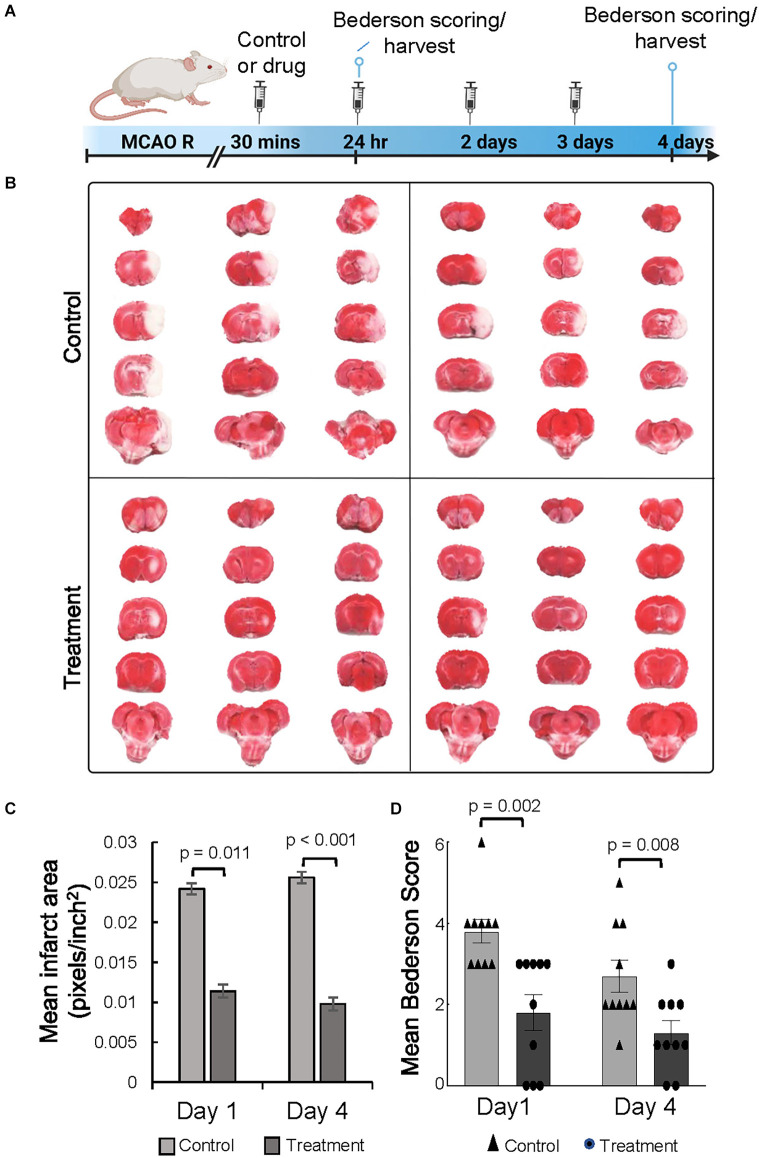
The SRC activator 10-1 attenuates cerebral ischemic injury and performance activity post-MCAO R. **(A)** Schematic representation of experimental procedures. Rats were injected with 10-1 (20 mg/kg) or control 30 min after a 90-min occlusion of the middle cerebral artery and every 24 h up to harvest at day 1 (*n* = 20) and day 4 (*n* = 20) after surgery. **(B)** Brains were harvested and stained with TTC to delineate and calculate infarct size. **(C)** Mean area of infarct was calculated at day 1 and day 4 post-MCAO R. **(D)** Neurological testing was done 24 h and 4 days after stroke onset using a modified Bederson score. *n* = 10 control and *n* = 10, 10-1.

To determine if 10-1 could ameliorate acute ischemic brain injury, both treatments were administered following reperfusion (Figure 1A). Infarct volume was assessed from TTC stained brain sections at 24 h and 4 days (Figures 1B,C). Overall, 10-1 showed significantly decreased infarct volumes compared to control at 24 h and 4 days (Figure 1C). Neurologic assessment indicated that when compared with the control group, 10-1 treated rats had significantly lower Bederson scores (Figure 1D) at 24 h and 4 days post-injury, indicating early and sustained protection against post-ischemia neurologic impairment.

### 10-1 treatment attenuates progression of cerebral tissue damage

**Figure 2:**
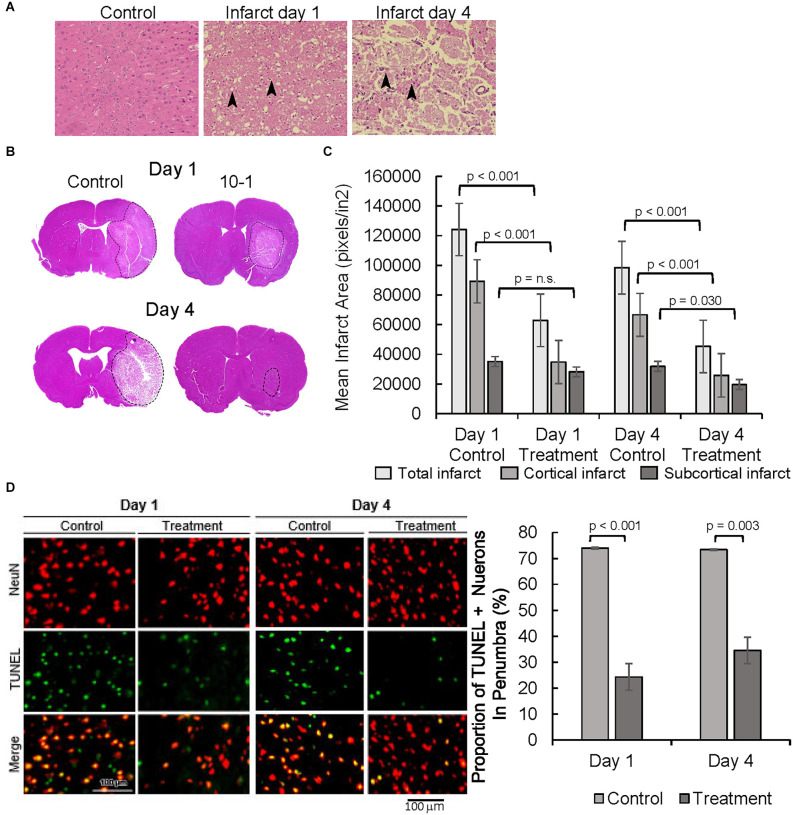
10-1 treatment attenuates progression of tissue damage post-MCAO R. **(A)** H&E tissue sections from the contralateral side (control, 1 day post-MCAO) and infarct areas at day 1 and day 4. Arrows indicate areas containing darkly stained pyknotic nuclei, cell body shrinkage, perineuronal vacuolization, and granular necrotic debris. **(B)** Representative H and E sections day 1 and day 4 post-MCAO. **(C)** Cortical and sub-cortical infarct size were measured in H&E stained brain tissue sections 1 day and 4 days post-MCAO R. **(D)** Representative images of TUNEL and NeuN immunostaining showing neuronal apoptosis one and 4 days post-MCAO R. Quantification of TUNEL positive NeuN positive cells. Day 1 and day 4 *n* = 10 control, 10 drug-treated, three images per section.

Following MCAO R, neuronal degeneration was determined by the presence of darkly stained pyknotic nuclei, cell body shrinkage, perineuronal vacuolization, and granular necrotic debris at days 1 and 4 (arrows in Figure 2A). Infarcted cerebral tissue is present in the cortex and subcortex (Figure 2B). Quantification of infarcted tissue in the cortex and subcortical structures revealed that 10-1 significantly decreased total infarct size and cortical infarct size at 24 h but not subcortical infarction (Figure 2C). On the other hand, 10-1 decreased cortex and subcortical infarct areas significantly at 4 days post-injury (Figure 2C). Thus, the damage is more severe in the subcortical tissue supplied by the proximal branches of the MCA (*e.g*., lenticulostriates), with fewer collaterals should the blood supply be interrupted. In contrast, cortical tissue seems to be less vulnerable to immediate cell death (*i.e*., necrosis) due to collateral circulation, and often represents at-risk tissue at early time points (*e.g*., the penumbra). Based on this data, 10-1 appears to lead to an attenuated progression of cerebral ischemic injury 24 h and 4 days post-injury (Figure 2B).

NeuN staining, a neuronal cell body-specific marker, and terminal deoxynucleotidyl transferase dUTP nick end labeling (TUNEL) assay, a measure of apoptotic activity, were then performed in the infarct penumbra (Figure 2D). The ischemic penumbra represents vulnerable but salvageable cerebral tissue (Ramos-Cabrer et al., 2011). Cells that are positive for NeuN and TUNEL represent apoptotic neurons. Overall, the portion of apoptotic neurons was significantly decreased in the penumbra of 10-1 treated animals at 24 h and 4 days post-injury (Figure 2D).

### 10-1 promotes anti-inflammatory microglia in the ischemic penumbra and Tregs in the ischemic core

**Figure 3:**
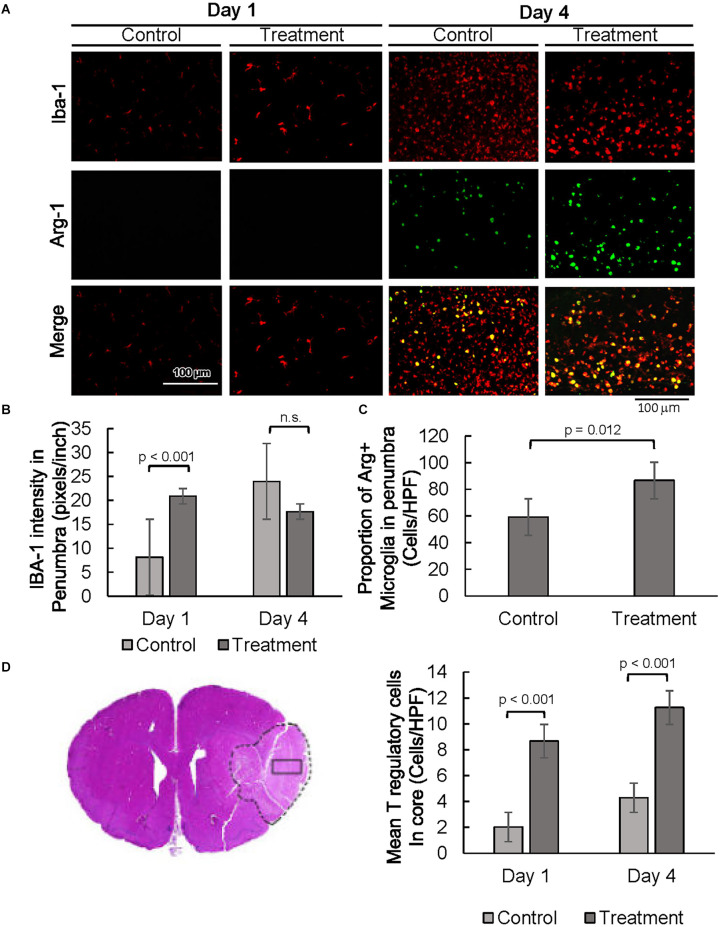
SRC activation promotes a pro-reparative immune response. **(A)** Representative images of IBA1 and ARG1 immunostaining showing per cent of pro-reparative M2 microglia 1 day and 4 days post-MCAO R. **(B)** Quantification of mean IBA1 intensity 1 and 4 days post-MCAO R. Day 1 and day 4 *n* = 10 control, 10 drug-treated, three images per section. **(C)** Quantification of IBA1 positive and ARG1 positive cells at day 4. **(D)** Quantification of average numbers of Tregs in the core infarct. Day 1 and day 4 *n* = 10 control, 10 drug-treated, three images per section.

Next, expression of the glial cell markers, ionized calcium-binding adaptor protein-1 (IBA1), CD86, and arginase-1 (ARG1) were evaluated. Resident microglia are characterized by small cell bodies, long-branched processes, and relatively low intensity of IBA1 staining (Figure 3 and Supplementary Figure 1A, Morizawa et al., 2017). In our subjects, IBA1 positive microglia in the penumbra of control animals 24 h post-injury were highly branched with elongated processes (Figure 3 and Supplementary Figure 1A). In contrast, microglial cells in the penumbra of 10-1-treated rats showed larger body sizes and had fewer branching processes, resembling active microglia (Davis et al., 2017). At day 4, differences in the activated-amoeboid phenotype in treated and control groups were less pronounced (Figure 3 and Supplementary Figure 1A).

Moreover, quantitative analysis shows a prominent increase in IBA1 expression in the penumbra of 10-1 rodents 24 h post-injury (Figures 3A,B). In addition, IBA1 expression was not increased in drug-treated animals as compared to controls on day 4, indicating an earlier peak in IBA1 expression in penumbral microglia (Figure 3B). Immunostaining was performed for CD86 to examine the effect of 10-1 on M1 microglia. We did not observe any changes in CD86 expression (data not shown).

We next investigated the expression of an alternative microglial activation marker, ARG1 (Munder et al., 1999; Cherry et al., 2014), specific to M2 microglia that is considered to be related to a more pro-reparative phenotype (Figures 3A,C) Co-localization of ARG1 and IBA1 was notably absent 24 h post-injury (Figure 3A). In contrast, expression of the ARG1 microglia marker was increased in IBA1 positive cells in 10-1 treated animals compared to control 4 days post-injury (Figures 3A,C). These results suggest that activated M2 microglia may contribute to improved recovery by day 4 after ischemic injury. Overall, SRC coactivator activation with 10-1 channels early post-stroke microglia activation into the more reparative M2 state during the later part of the post-injury response.

Lastly, we had recently shown enrichment of SRC-3 expression in regulatory T cells (Tregs; Nikolai et al., 2021). Given the protective effects of Tregs in post-stroke outcomes in other studies (Zhang et al., 2021), we performed immunostaining for Foxp3, a specific nuclear marker for Tregs (Figure 3D and Supplementary Figure 1B). Foxp3 staining was exclusive to the core, indicating Tregs are absent in the penumbral region 24 h and 4 days after injury. Foxp3 expression was elevated in the infarct core in 10-1 group as compared with controls at 24 h and 4 days following injury (Figure 3D).

### 10-1 treatment attenuated astrogliosis and promoted oxidative stress protection in astrocytes

**Figure 4:**
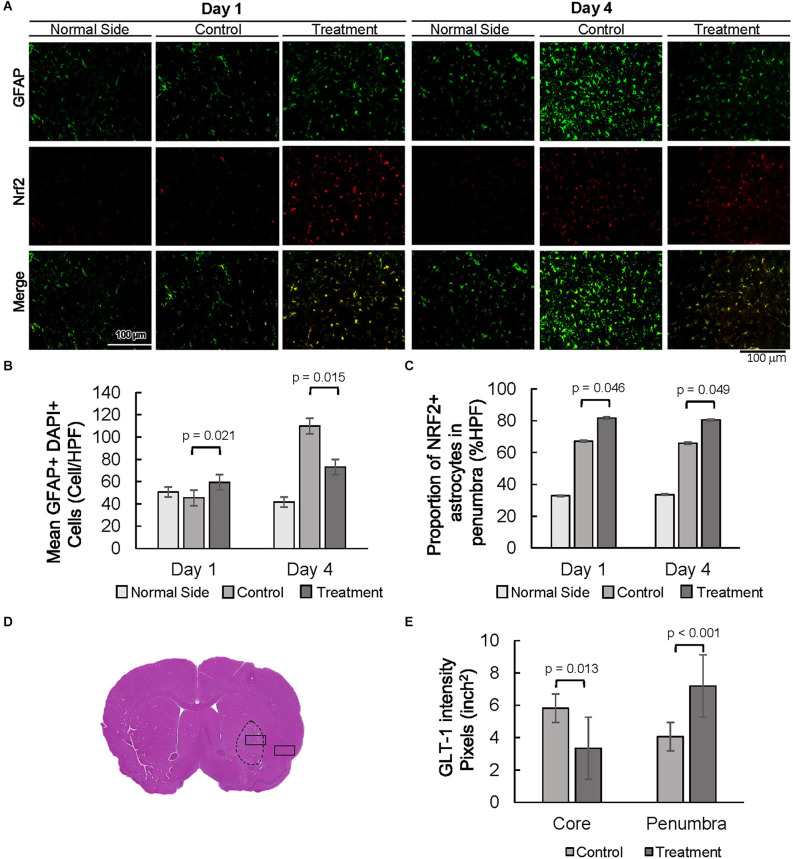
Decreased astrogliosis is associated with an increased proportion of NRF2 and GLT-1 positive astrocytes. **(A)** Representative images of GFAP and NRF2 in the contralateral side (normal side) and penumbra 1 day and 4 days post-MCAO R. **(B)** Number of astrocytes in the penumbra. **(C)** The proportion of GFAP positive, NRF2 positive astrocytes in the penumbra. **(D)** Representative H&E section with area of sampling in core (box inside dashed area) and penumbra (box outside dashed area). **(E)** Quantification of the intensity of GLT-1 staining in the core and penumbra as shown in Supplementary Figure 2. Day 1 and day 4 *n* = 10 control, 10 drug-treated, three HPF per section.

Expression of astrocyte-specific glial fibrillary acidic protein (GFAP) was evaluated as a marker of astrogliosis in the stroke penumbra (Figure 4A). Quantitative GFAP analysis showed that GFAP expression at 4 days post-injury was significantly decreased in the stroke penumbra of 10-1 group as compared to controls, suggesting an attenuation in astrogliosis (Supplementary Figure 2A, Yang and Wang, 2015). On the other hand, analysis of the number of GFAP positive cells showed that the number of astrocytes in the penumbra of 10-1 animals was slightly increased from controls and was not significantly different from the contralateral normal side of the brain at 24 h (Figures 4A,B), suggesting a relative preservation of astrocytes. As expected, by day 4, GFAP positive cells were significantly increased in the stroke penumbra of control animals, beyond the mean number in the 10-1 group or contralateral normal side (Figure 4B). Quantitative analysis of GFAP positive cells showed that the number of astrocytes was significantly decreased in the penumbra of 10-1 treated animals compared to controls (Figure 4B).

The oxidative stress marker NFE2 Like BZIP Transcription Factor 2 (NFE2L2/NRF2) was examined next, as a measure of antioxidant protection in the stroke penumbra (Figure 4A). Overall, NRF2 expression was increased in the stroke penumbra of 10-1 subjects 4 days post-injury, but not significantly at 24 h (Supplementary Figure 2B). However, co-staining of GFAP and NRF2, a measure of astrocyte-specific expression of NRF2, showed increased co-expression at 24 h and 4 days post-injury (Figure 4C).

Lastly, we investigated the expression of astrocytic solute carrier family 1 member 2 (SLC1A2, also known as glutamate transporter glutamate transporter-1, GLT-1) as a marker of excitotoxic astrocytic cell death (Fontana, 2015). GLT-1 expression is well described in astrocytes in response to ischemic injury, peaks at 24–48 h, and dissipates rapidly after 4 days (Peterson and Binder, 2019). Quantification of GLT-1 staining intensity at 24 h post-injury suggests an increase in GLT-1 expression in penumbral astrocytes compared to the core infarct (Figures 4D,E and Supplementary Figure 2B).

## Discussion

Neuroprotection is an important unmet need in the management of AIS. In this study, we have shown that SRC activation with 10-1 provides protection against cerebral injury following ischemia and reperfusion. In our model, infarct volumes were decreased by over 50% after treatment as compared with control animals and neurologic function remained relatively preserved. We showed that this effect most likely represents protection from ischemia rather than tissue regeneration. Furthermore, our analysis of neuronal apoptosis in the ischemic penumbra of 10-1 treated subjects was likewise decreased by over 50%. As SRCs are highly expressed in the brain and coordinate the activation of multiple gene expression programs necessary for cerebral repair (Sun and Xu, 2020), we hypothesized that this apparent neuroprotection could be explained by changes in the phenotypes of immune and glial cells.

Our findings indicate that 10-1 activates microglia to take on a more pro-reparative, M2 phenotype, that can help attenuate neurodegeneration in response to ischemic injury. Studies have shown that the presence of infiltrating macrophages from the peripheral circulation is low on the first day after stroke (Schilling et al., 2003). Thus, our data is highly suggestive that the increase in IBA1 expression we have noted is most likely a result of resident microglia activation. M2 microglia secrete anti-inflammatory cytokines that promote neuronal growth and are considered to be overwhelmingly neuroprotective (Deng et al., 2020). Nuclear receptors have well-described actions in modulating inflammatory processes (Glass and Ogawa, 2006). SRC-3 expression prevents NF-κB activation and protects against lymphoma formation in mice, indicating that SRC-3 can function to suppress the innate immune response (Wu et al., 2002; Coste et al., 2006). While our previous studies show that SRC activation suppresses inflammatory signaling (Coste et al., 2006; Yu et al., 2007; Chen Q. et al., 2010; Mullany et al., 2021), these results suggest 10-1 also provides neuroprotection by promoting early microglial activation in the acute tissue response (up to 48 h after stroke). We previously reported SRC activation suppressed macrophage inflammatory signaling and promoted M2 reparative macrophages in ischemic hearts (Mullany et al., 2020). In the current study, we found that early microglial activation is followed by the polarization of M2 microglia in the subacute phase (3–6 days after stroke), which may ultimately be contributing to the robust neuroprotection that we see. Although these findings are consistent with previously documented SRC actions, further research is needed to identify the receptors and signaling pathways involved in 10-1’s regulation of microglial functions.

Ongoing inflammation in the brain following stroke is a major contributor to tissue damage (Chamorro et al., 2012). Animal studies show that Tregs can suppress inflammation and promote recovery in both the acute and chronic phases (Zhang et al., 2021). In this study, we show an increased number of Tregs at 24 h and 4 days in the post-stroke infarct core. Other animal studies have shown that Tregs migrate to the ischemic infarct core 7–14 days after stroke onset (Ito et al., 2019). Treg functions have been implicated in many neuroprotective processes, such as the suppression of astrogliosis by producing amphiregulin and the promotion of reparative microglia by secretion of osteopontin (Shi et al., 2021). Recently, we discovered that SRC-3 expression is enriched in Tregs, and that inhibition of SRC-3 blocks their immunosuppressive functions (Nikolai et al., 2021). These findings reveal another mechanism by which SRC activation in AIS may prevent ongoing inflammation, supporting improved tissue recovery.

While microglia and other immune cells comprise a relatively small population of cells in the brain, glia are even more numerous than neurons. Astrocytes are a large part of the glial population, especially in the cortical regions of the brain, and perform multiple homeostatic functions necessary for proper cerebral function and maintenance (Pajarillo et al., 2019). Astrocytes function to modulate synaptic function, promote angiogenesis, and provide antioxidant protection (Becerra-Calixto and Cardona-Gomez, 2017). However, similar to microglia, astrocytes are not fate-arrested and can alter their morphology and function in response to environmental cues (Liddelow and Barres, 2017). Astrocyte glutamate transport by GLT-1, responsible for over 90% of synaptic clearance of excess glutamate, is critical for neuronal survival following cerebral ischemia (Pajarillo et al., 2019). Thus, an increase in astrocyte GLT-1 expression in the stroke penumbra of 10-1 treated animals suggests that GLT-1 is a key player in attenuating neuronal cell death and infarct progression.

In addition, oxidative stress-induced cerebral damage is an important mechanism of injury potentiation in ischemic stroke. NRF2 serves as the transcriptional master regulator of basal and stress-induced cytoprotective responses in the brain and in many other organs (Liu et al., 2019). Furthermore, NRF2 is highly expressed in astrocytes (Shih et al., 2003), where previous neuroprotection studies have implicated NRF2-regulated genes as the likely target of neuron–astrocyte interactions after injury (Habas et al., 2013). Increased NRF2 expression in astrocytes from 10-1 treated animals supports the notion that astrocytes are major contributors to oxidative stress management in response to stroke. In addition, higher expression of NRF2 in stroke penumbral astrocytes at 4 days post-MCAO R, suggests a higher propensity for 10-1 conditioned astrocytes to adapt and survive when exposed to oxidative stress. Previous research has previously shown that SRC-3 is required for transcriptional activation of the anti-oxidative enzyme catalase (CAT; Chen Q. et al., 2010) and is recruited to the promoters of CAT (Chen Q. et al., 2010) and NRF2 (Kim et al., 2013). This suggests SRC-3 activation contributes directly to the regulation of astrocyte antioxidant signaling pathways. These findings suggest that 10-1 may contribute to early and sustained neuroprotection by promoting the NRF2 and GLT-1 astrocyte responses, which may work to attenuate neuronal excitotoxicity and oxidative injury. Taken together, these findings indicate that 10-1 treatment promoted early microglial activation along with reparative microglia and increased Tregs. 10-1 also improved astrocyte oxidative stress responses and attenuated astrogliosis.

While we believe that the results of this study are robust and represent a breakthrough in neuroprotection research, we do appreciate several limitations. Firstly, the phenotypic changes seen in neurons, microglia, and astrocytes do not provide complete insight into gene expression. Further detailed studies are required to provide insights into the transcriptional regulation of anti-inflammatory microglial cells and the pro-survival oxidative responses of astrocytes. Ultimately, understanding the differential gene expression programs will help us to clarify what cell types are most implicated in the neuroprotective effects of 10-1, which will help us identify the drug’s optimal use in AIS. Additionally, although the concern for sex differences with 10-1 therapy in the current study is eliminated, the fact that we only employed male rats for our tests is a weakness in our findings. For example, in another rodent study of the central nervous system, there was an increased expression of SRC-1 in female mice after spinal cord injury, which lead to better neurologic recovery than in male mice (Xiao et al., 2017), emphasizing the importance of future studies using female rodents.

**Figure 5:**
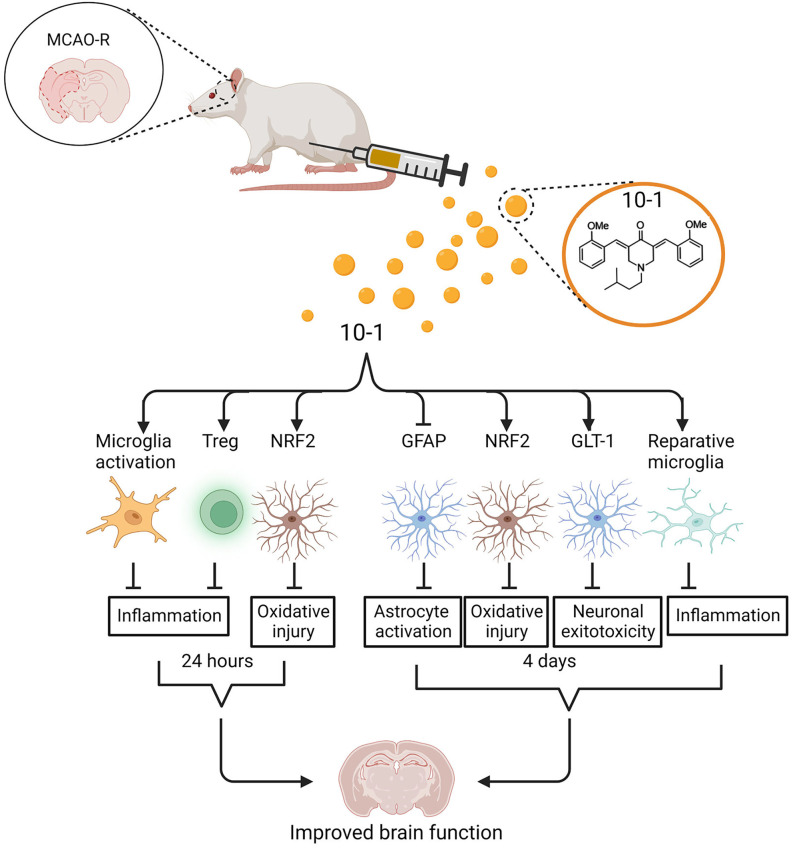
Schematic of regulation of ischemic stroke injury following 10-1 treatment. 10-1 treatment attenuated neuronal injury following robust transient activation of microglia, increased Treg numbers, and increased representation of NRF2 positive astrocytes 24 h after ischemic injury. Four days after ischemia injury, the 10-1 therapy also enhanced NRF2 positive astrocytes, polarized M2 reparative microglia, and reduced astrogliosis. Created with BioRender.com.

We demonstrate that SRC-coactivator activation with MCB-10-1 provides extensive neuroprotection in a mouse MCAO R model through a coordinated, multicellular process, which is consistent with previously documented SRC activities in tissue formation and homeostasis. We have shown that not only are infarct sizes and neurologic function improved in the 10-1 treatment group but that the secondary injury response of inflammatory cells and astrocytes also is attenuated. 10-1 improved recovery after MCAO R in the acute phase, and was associated with increased M2 microglial activation, increased astrocyte NRF2 and GLT-1 expression, and increased Treg cell numbers recruited into the stroke penumbra (Figure 5). In addition to attenuating damage from injury, SRC stimulation with 10-1 could provide a potential therapeutic approach to extend the therapeutic window for revascularization strategies. In summary, SRC activation may be able to fill the unmet clinical need for an effective neuroprotectant in stroke patients.

## Data Availability Statement

The raw data supporting the conclusions of this article will be made available by the authors, without undue reservation.

## Ethics Statement

The animal study was reviewed and approved by Institutional Animal Care and Use Committee, University of Texas Medical Branch at Galveston.

## Author Contributions

BO’M, LM, CD, DL, RG, and PK: conceptualization. LM: writing—original draft. LM, DL, BO’M, CD, PK, RG, AR, VV, ASR, ZL, AS, and TL: investigation, methodology, and formal analysis. BO’M, PK, and DL: resources. LM, DL, BO’M, CD, PK, RG, and TL: writing—review and editing. RG and TL: data curation and visualization. All authors contributed to the article and approved the submitted version.

## Funding

This work was supported by NSF: NeTS 1801865, Philip J. Carroll, Jr. Professorship, Brockman Foundation, Rene and Kay Joyce Family Foundation, Sonya T. and William A. Carpenter, Jr. to CD.
