# Supplementary figures and images for: A steroid receptor coactivator small molecule “stimulator” attenuates post-stroke ischemic brain injury
Source: Front Mol Neurosci. 2022 Dec 1;15:1055295. doi: 10.3389/fnmol.2022.1055295 (PMC9751323; doi:10.3389/fnmol.2022.1055295)

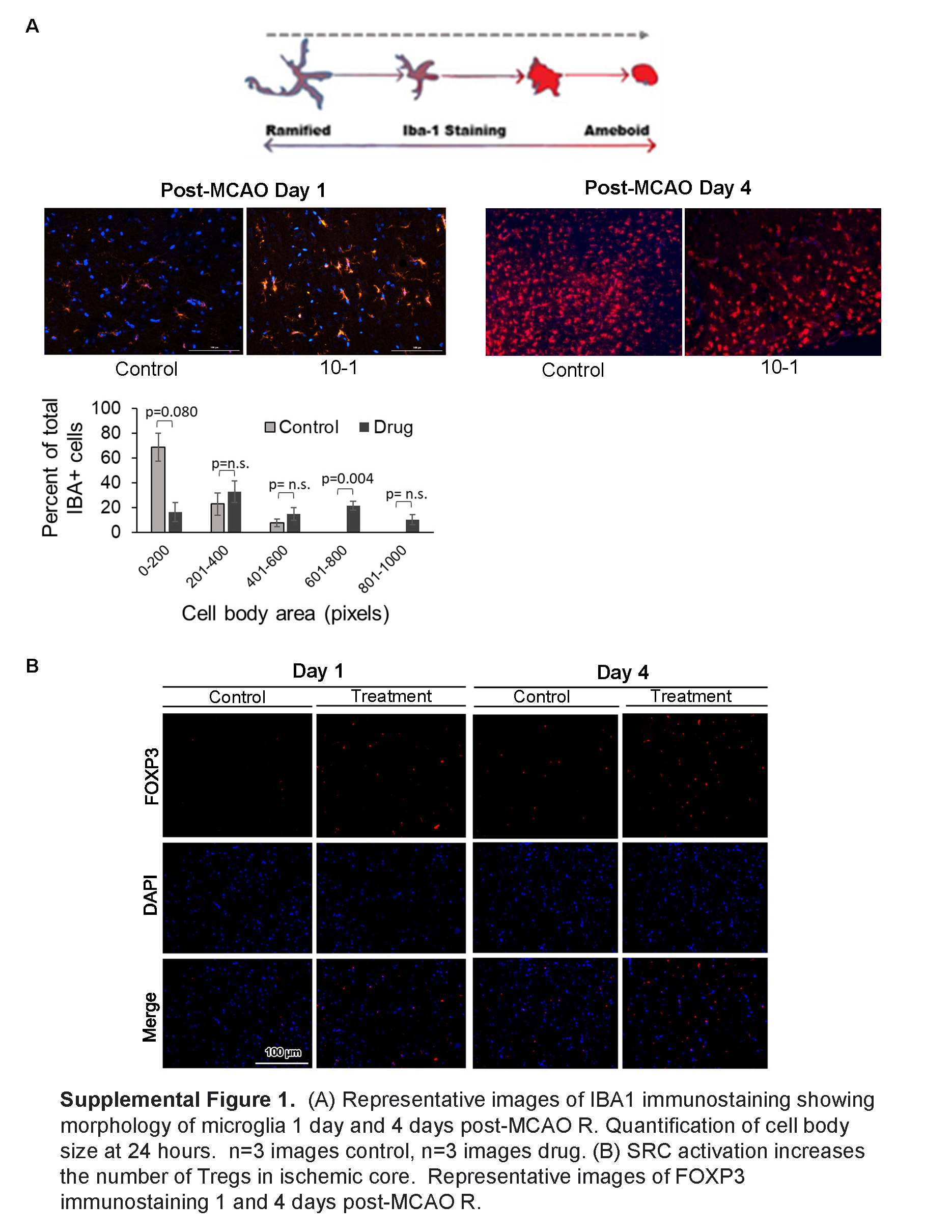

Supplement: Supplementary file 1 [file Image_1.TIF]

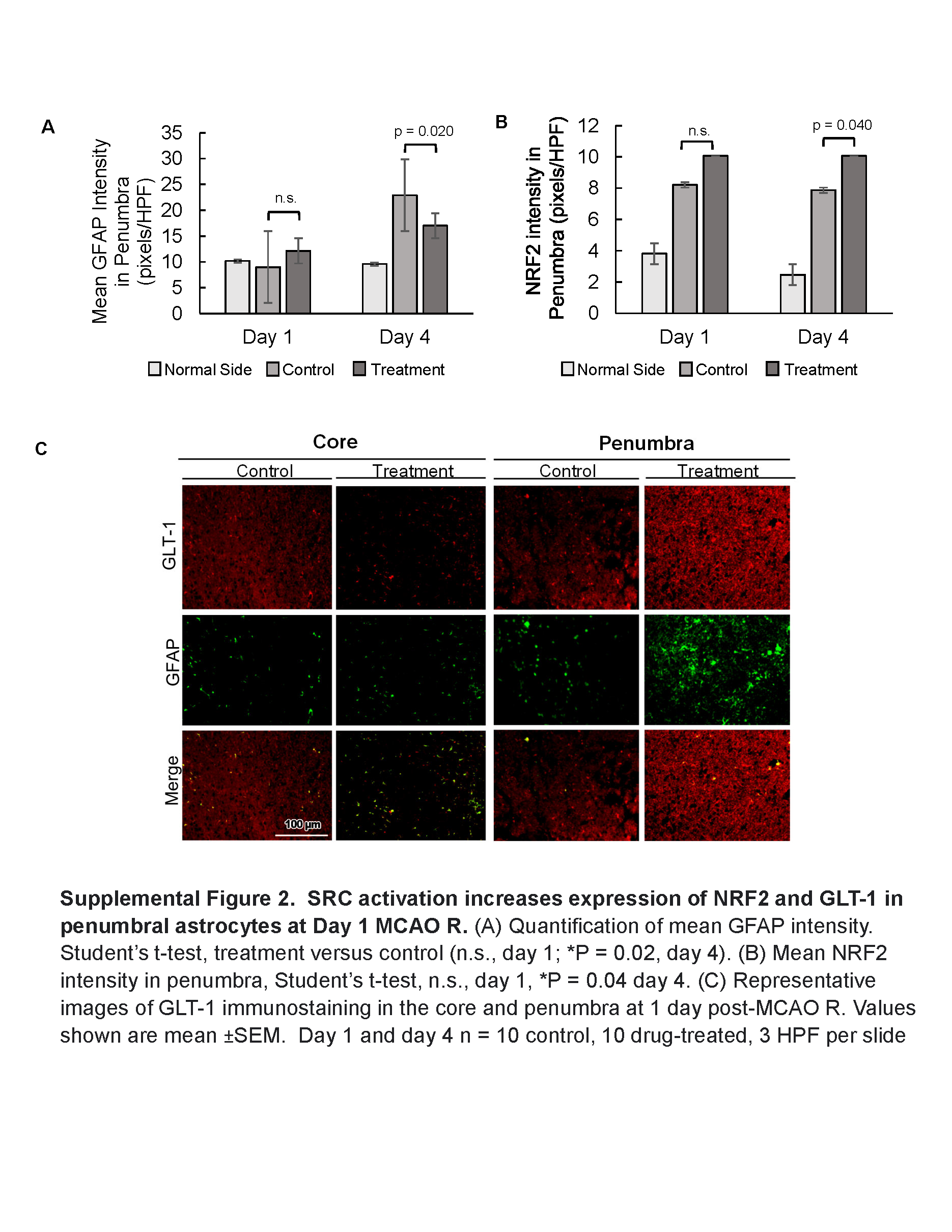

Supplement: Supplementary file 2 [file Image_2.TIF]
